# Idiopathic pulmonary fibrosis and diabetes mellitus: a meta-analysis and systematic review

**DOI:** 10.1186/s12931-021-01760-6

**Published:** 2021-06-08

**Authors:** Le Bai, Li Zhang, Tingyu Pan, Wei Wang, Dian Wang, Cassidy Turner, Xianmei Zhou, Hailang He

**Affiliations:** 1 Affiliated Hospital of Nanjing University of Chinese Medicine, Nanjing, 210029 China; 2 Department of GCP Research Center, Jiangsu Province Hospital of Chinese Medicine, Nanjing, 210029 China; 3 Arizona Metabolomics Laboratory, College of Health Solutions, Arizona State University, Scottsdale, AZ USA; 4 Department of Respiratory Medicine, Jiangsu Province Hospital of Chinese Medicine, 155 Hanzhong Road, Nanjing, 210029 Jiangsu Province People’s Republic of China

**Keywords:** Idiopathic pulmonary fibrosis, Diabetes mellitus, Meta-analysis, Systematic review

## Abstract

**Background:**

Idiopathic pulmonary fibrosis (IPF) is a chronic diffuse interstitial lung disease, of which the etiology has been poorly understood. Several studies have focused on the relationship between IPF and diabetes mellitus (DM) in the past years but have failed to reach a consensus. This meta-analysis aimed to examine the association between diabetes to IPF.

**Methods:**

We accumulated studies investigating the association between DM and IPF from databases including Medline, Cochrane Library, Embase, Web of Science, and China National Knowledge Infrastructure. RevMan 5.3 and the Newcastle–Ottawa Scale (NOS) were utilized to analyze the data and assess the quality of the included studies. The value of odds ratio (OR) with 95% confidence interval (CI) was used as the measure to estimate the risk of DM in IPF. Heterogeneity was assessed by *I*^2^ statistics. We also performed subgroup analysis, meta-regression, and Egger’s test for bias analysis.

**Results:**

Nine case–control studies with 5096 IPF patients and 19,095 control subjects were included in the present meta-analysis, which indicated a positive correlation between DM and IPF (OR 1.65, 95% CI 1.30–2.10; *P* < 0.0001). Meta-regression and subgroup analysis negated the influence of covariates like cigarette smoking, age and gender, but the heterogeneity existed and could not be fully explained.

**Conclusion:**

IPF and DM may be associated, but the causal relationship remains indeterminate till now. Further rigorously designed studies are required to confirm the present findings and investigate the possible mechanisms behind the effect of DM on IPF.

## Introduction

Idiopathic pulmonary fibrosis (IPF) is a progressive and fatal pulmonary disease with an annual cumulative prevalence of 18.2 cases per 100,000 persons in America [1] while a median survival of only 3–5 years [2, 3]. IPF is characterized pathologically by proliferation and differentiation of lung fibroblasts. Cigarette smoking, age and gender, chronic viral infections, etc., are considered as risk factors of IPF [4]. In the past decade, the understanding of IPF pathogenesis has shifted from an inflammatory-driven process [5] to the hypothesis of aberrant activation of the alveolar epithelial cells [6]. Nevertheless, the exact etiology of IPF remains unclear.

In recent years, it has been observed that IPF patients are often additionally diagnosed with diabetes mellitus (DM), resulting in increased research interest in the correlation between these two diseases. Firstly, two earlier case-controls studies [7, 8] were successively conducted in Japan but obtained opposite conclusions. Subsequent clinical observational studies [9, 10] suggested that DM was likely to increased the risk of IPF, which inspired experiments related to antidiabetic treatment for IPF. Interestingly, using mice animal models, it was revealed that metformin could reverse established lung fibrosis [11–15], however, a post hoc analysis [16], which investigated the effect of combinations therapy in IPF (pirfenidone/pirfenidone + metformin), concluded that metformin had no effect on clinically relevant outcomes. Furthermore, another pooled analysis demonstrated that metformin might increase the risk of disease progression when in combination with proton pump inhibitors, angiotensin II receptor blockers or thyroid medications [17].

Due to the conflicting results in the existing studies, it remains controversial whether DM is truly correlated with IPF. Hence, in the present study, we conducted a meta-analysis and systematic review, aiming to assess the association between DM and the incidence of IPF.

## Materials and methods

We performed meta-analysis and wrote this report referring to the Meta-analysis Of Observational Studies in Epidemiology (MOOSE) proposal [18] and the Preferred Reporting Items for Systematic Reviews and Meta-Analyses (PRISMA) statement [19].

### Literature search

We searched databases including Medline, Cochrane Library, Embase, Web of Science and China National Knowledge Infrastructure. The following items were searched in databases as keywords or random words: “pulmonary fibrosis”, “diabetes”, “risk factors” and such searches were additionally filtered for articles published in any language leading up to September 30, 2020 (Complete search strategy presented in Appendix 1).

### Inclusion criteria and exclusion criteria

Case–control studies or cohort studies were selected. The case groups were all diagnosed with IPF in accordance with clinical history, High-Resolution Computed Tomography (HRCT), and when available, lung biopsy. Also, a calculated measure of association between DM and IPF was required. Studies focusing on progression or prognosis of IPF and studies lacking general information about control groups were excluded.

### Data extraction

Two researchers (L.B. and L.Z.) managed data extraction independently, reviewing the title, abstract, and full text of each article, and discussed or consulted a third researcher (T.P.) when disputations arose. The following are included: (1) basic information of each study including author, publication year, study design, etc.; (2) characteristics of case and control groups; (3) diagnostic methods of DM and IPF; (4) the number of diabetics in case and control groups; and (5) potential sources of biases.

### Quality assessment

The Newcastle–Ottawa Scale (NOS) was used for quality assessment of included studies, covering three domains: selection of groups, comparability of groups and ascertainment of exposure [20]. The NOS score ranges from 0 to 9 stars and studies that receive 5 stars or more are regarded as high quality. We evaluated the diagnostic criteria of IPF and DM in each study for the possibility of selection bias. Cigarette smoking, age, gender, environmental exposure and genetic factors, which may induce IPF and bring about information bias, were deemed as covariates and all taken into consideration when estimating whether control subjects were adequately selected.

### Statistical analysis

In our meta-analysis, odds ratio with 95% confidence interval (95% CI) was used as the effect measure. Heterogeneity was assessed by *I*^2^ statistics and random effect model was chosen when heterogeneity was significant (*I*^2^ > 50%), otherwise, fixed effect model was selected. Forest plots were used to display the results from individual studies and pooled estimates, and *P* < 0.05 were regarded as statistically significant. Trial Sequential Analysis (TSA) was used for estimate of evidence size and reliability of the conclusion [21, 22]. We also performed sensitivity and subgroup analyses to assess resources of heterogeneity. Meta-regression and Egger’s test [23] were utilized for bias analysis. Data analysis was performed using RevMan 5.3, Stata 12, and TSA 0.9 beta.

## Results

### Study selection and characteristics

**Fig. 1:**
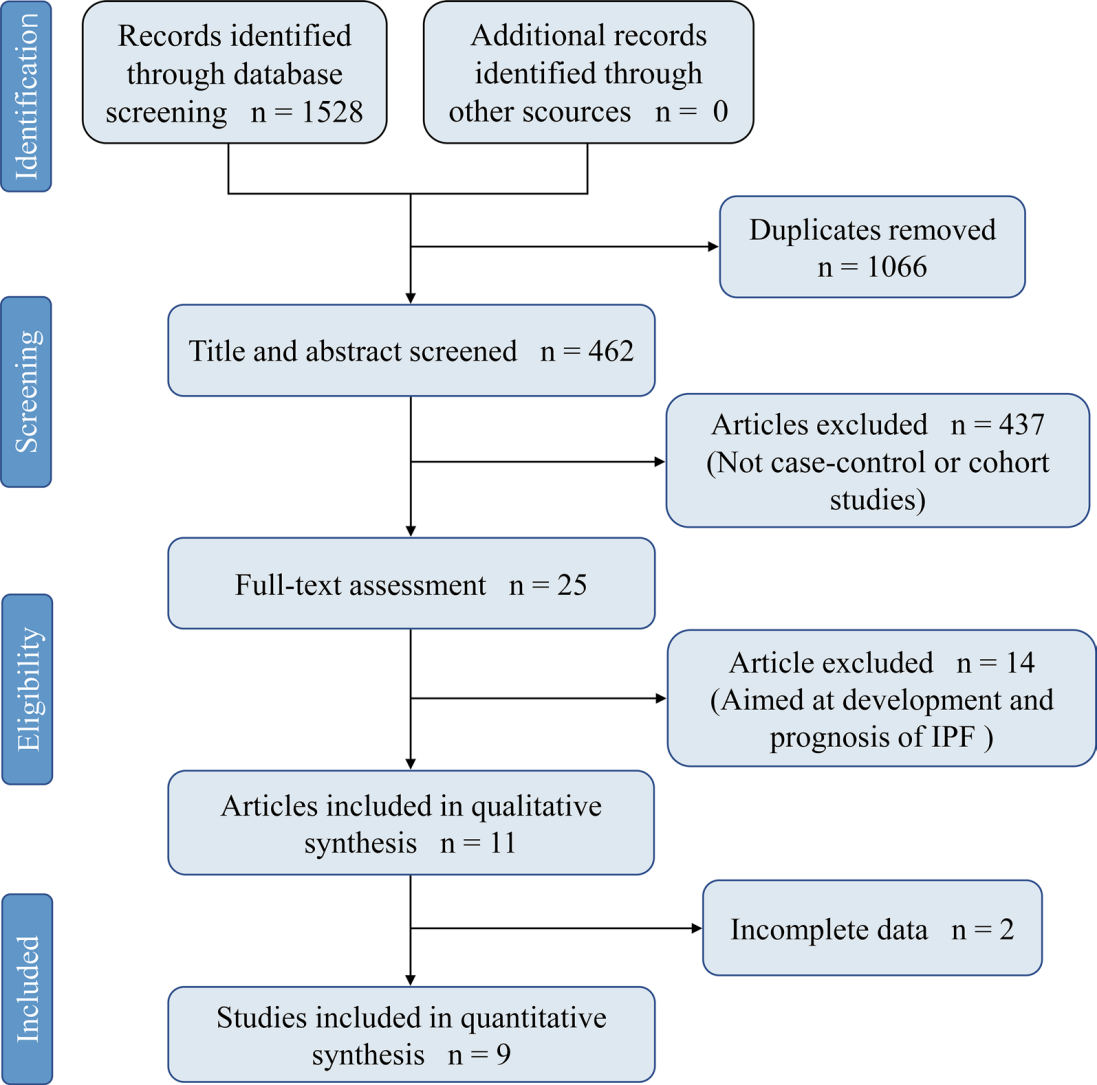
The flow diagram of study selection

As is briefly illustrated in Fig. 1, out of the 1528 articles reviewed, 9 studies [7–10, 24–28] from 5 countries finally met our eligibility. All studies were case–control and distinguished as high-quality by NOS assessment. General population was selected as control groups in six studies [7, 8, 10, 25–27], one study included only healthy volunteers [24], and the remaining two [9, 28] included patients with other chronic pulmonary diseases. IPF was diagnosed based on clinical history, HRCT, and lung biopsy while diagnosis of DM could be established with any objective method such as fasting blood glucose or simple by clinical symptoms combined with clinical history. More details are displayed in Table 1.

**Table 1:** Characteristics of Included Studies

Study	Country	Design	NOS	IG	CG	Method of IPF Diagnosis	Method of DM Diagnosis
Enomoto et al. [7] 2003	Japan	Case–Control	8	52	184 people matched for age and sex with no lung disease by chest radiographs	ATS/ERS criteria [66]	FBG > 6 mmol/L and/or HbAIc > 6% in combination with any treatment history
Miyake et al. [8] 2005	Japan	Case–Control	8	104	56 acute bacterial pneumonia, and 4 common cold, matched by age and sex	ATS/ERS criteria [66]	Medication or diet treatment history
Gribbin et al. [10] 2009	United Kingdom	Case–Control	6	920	3593 control subjects matched by age, gender and general practice	Read Code (diagnostic terms) in THIN database	Read Code
Ma.C et al. [9] 2010	Mexico	Case–Control	6	97	560 patients, 461 with other pulmonary diseases and 98 with otorhinolaryngologic problems	ATS/ERS criteria [66]	FPG > 6 mmol/L. Clinical history and medication therapy were also referred to
Garcia-Sancho et al. [24] 2011	Mexico	Case–Control	8	100	263 healthy control subjects matched for age, sex, and place of residence	ATS/ERS criteria [66]	Clinical symptoms and medication history
Kim et al. [25] 2015	Korea	Case–Control	7	460	1925 control subjects matched with age, gender, and smoking habits	ATS/ERS/JRS/ALAT criteria [65]	FPG > 6 mmol/L together with clinical history
Dalleywater et al. [27] 2015	United Kingdom	Case–Control	8	3211	12,307 control subjects, matched for age, sex, and general practice	A new diagnosis prior to previous Read Code	Read Code
Zhong et al. [28] 2016	China	Case–Control	6	108	115 patients without respiratory failure or other underlying disorders	Guidance for Diagnostic and Treatment of Pulmonary Fibrosis (Chinese Thoracic Society 2002)	FPG > 6 mmol/L and/or 2-h PG > 11.1 mmol/L
Xu et al. [26] 2020	China	Case–Control	7	44	88 patients without evidence of lung disease on computed tomography, matched for age and sex	ATS/ERS criteria [4]	FPG > 7 mmol/L. Clinical history was also used for diagnosis

NOS: Newcastle–Ottawa Scale. IPF: Idiopathic Pulmonary Fibrosis. DM: Diabetes Mellitus. IG: IPF Group. CG: Control Group. THIN: The Health Improvement Network. FBG: Fasting Blood Glucose. FPG: Fasting Plasma Glucose. ATS: American Thoracic Society. ERS: European Respiratory Society. JRS: Japanese Respiratory Society. ALAT: Latin American Thoracic Association

### Meta-analysis

**Fig. 2:**
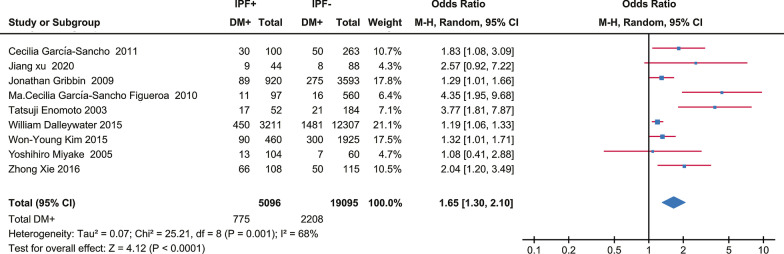
Forest plot of individual and pooled effect of odds ratio for DM in IPF. *IPF* idiopathic pulmonary fibrosis, *DM* diabetes mellitus, *M–H* Mantel–Haenszel

**Fig. 3:**
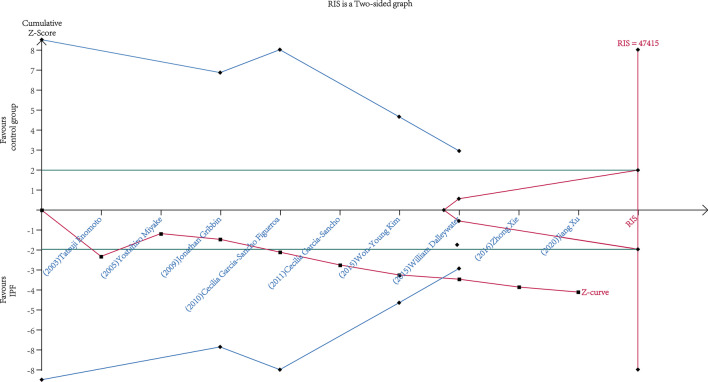
Trial sequential analysis for the 9 studies providing the calculation of correlativity between DM and IPF. The diversity-adjusted required information size (RIS) was 47,415 patients, with α = 5% (two-side), power of 80%, relative risk reduction of 35% and incidence of 3% in the control arm. The cumulative Z curve got across both the conventional boundary (green line) and the trial sequential monitoring boundary (blue line), indicating that the information size was sufficient and the outcome was reliable

**Fig. 4:**
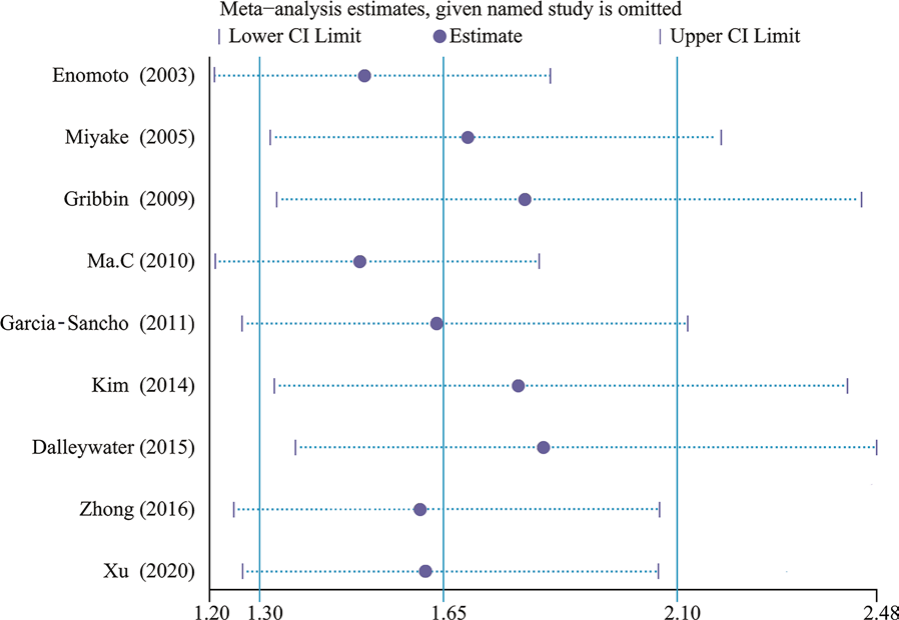
Sensitivity analyses of the primary meta-analysis. Every odds ratio was located between 1.30 and 2.10 while none of 95% confidence intervals crossed the invalid line “1”, signifying that the result was stable

A total of 5096 IPF cases and 19,095 control subjects were involved in the analysis (Fig. 2), which suggested a significant association between DM and IPF (OR 1.65, 95% CI 1.30–2.10; *P* < 0.0001), based on statistical reliability verified by the subsequent trial sequential analysis (Fig. 3). The heterogeneity was significant (*I*^2^ = 68%) with no obvious sources of biases found among the sensitivity analyses (Fig. 4). Therefore, we performed subgroup analyses to investigate factors that possibly contribute (Table 2). The result remained consistent in separate analyses regardless of community or hospital controls, and irrespective of diagnostic criteria of IPF/DM or characteristics of control groups (healthy subjects, general population or patients with pulmonary disorders). When smoking status, age, and gender were accounted for in case and control groups, the heterogeneity still existed as before.

**Table 2:** Subgroup analysis

Study characteristic	Study	IPF (D/T)		Control Group (D/T)		OR	95%CI	*P* value	Heterogeneity
Groups matched by age and sex [7, 8, 10, 24–27]	7	698	4891	2142	18,420	1.43	1.16–1.76	*P* = 0.0007	*I*^2^ = 55%
Smoking status matched in both groups [8–10, 25]	4	203	1581	598	6138	1.52	1.05–2.19	*P* = 0.02	*I*^2^ = 65%
Control groups made up of general population [7, 10, 25–28]	6	721	4795	2135	18,212	1.49	1.19–1.88	*P* = 0.0007	*I*^2^ = 66%
Control group with pulmonary diseases [8, 9]	2	24	201	23	620	2.23	0.56–8.88	*P* = 0.25	*I*^2^ = 79%
Healthy control group [24]	1	30	100	50	263	1.83	1.08–3.09	…	…
Community controls [7, 24]	2	47	152	71	447	2.50	1.24–5.06	*P* = 0.01	*I*^2^ = 59%
Hospital controls [8–10, 25–28]	7	728	4944	2137	18,648	1.47	1.17–1.84	*P* = 0.008	*I*^2^ = 61%
Diagnosis of IPF based on ATS/ERS criteria [7–9, 24–26]	6	170	857	402	3080	2.09	1.34–3.29	*P* = 0.001	*I*^2^ = 66%
Diagnosis of DM based on FBG [7, 9, 25, 26, 28]	5	193	761	395	2872	2.38	1.43–3.97	*P* = 0.0009	*I*^2^ = 72%
Diagnosis of DM based on subjective methods [8, 24]	2	43	204	57	323	1.62	1.02–2.58	*P* = 0.04	*I*^2^ = 0

D: Diabetes. T: Total. IPF: Idiopathic Pulmonary Fibrosis. DM: Diabetes Mellitus. FBG: Fasting Blood Glucose. ATS: American Thoracic Society. ERS: European Respiratory Society

### Bias analysis

**Fig. 5:**
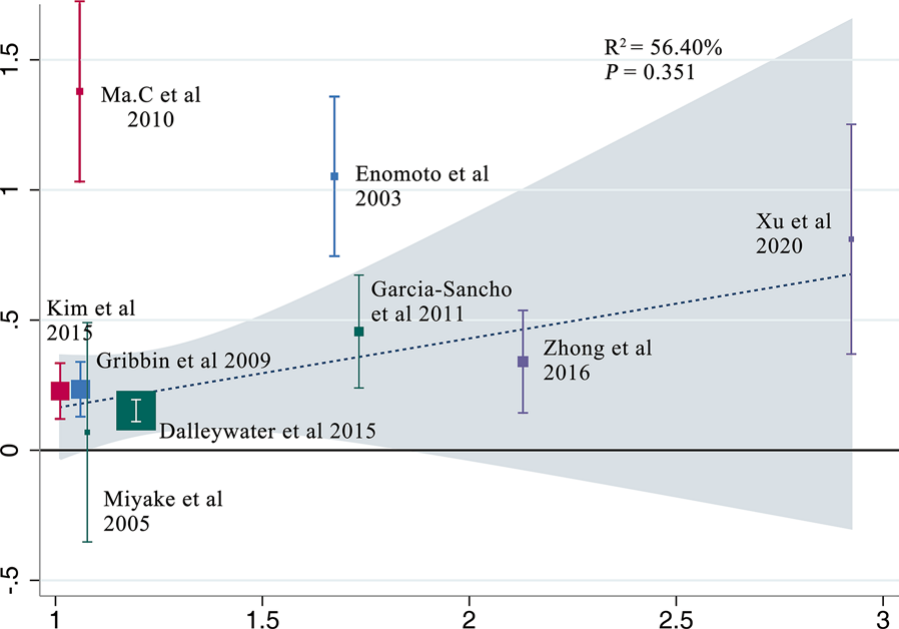
Meta-regression analysis presenting the impact of smoking on relevance between DM and IPF. The X-axis represents the ratio of proportion of smokers in IPF group to that in control group in each study (this was used to represent the influence of smoking in each study), while the Y-axis accordingly represents the ratio of the morbidity of DM in IPF group to that in control group (this was used to represent the association between DM and IPF). In the meta-regression analysis, no significant linear correlation was found (P = 0.351 > 0.05) between Y-axis and X-axis, which indicated that smoking didn’t affect the association between DM and IPF

**Fig. 6:**
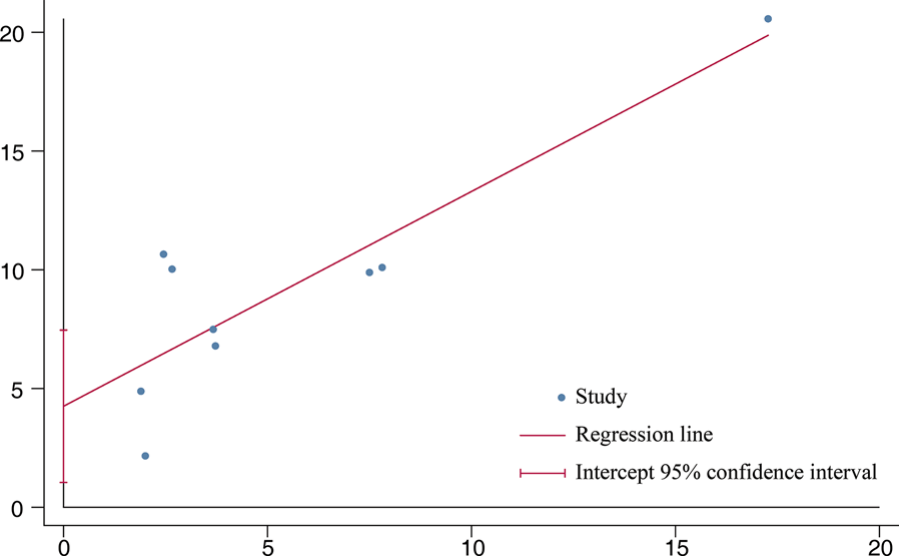
Egger’s publication bias plot for included studies

All elements that could lead to IPF were considered as potential sources of biases. Firstly, cigarette smoking is a known risk factor for IPF [29] and in the five included studies [7, 24, 26–28], smokers or ex-smokers were much more in case groups than in controls. In the following subgroup analysis (Table 2), when we selected the other four studies [8–10, 25] in which the smoking habits between case and control groups were similar, the association remained statistically significant (OR 1.52, 95% CI 1.05–2.19; *P* = 0.02), which coincided with the outcome of the meta-regression (Fig. 5, *P* = 0.351), suggesting that smoking was unlikely to distort the final results. Next, considering that age and gender are related to IPF despite inexplicable reasons [4], seven studies [7, 8, 10, 24–27] in which these two factors were well-balanced were used to inform another analysis, yet again concluding that DM correlated with IPF (OR 1.43, 95% CI 1.16–1.76; *P* = 0.0007). Still, there remained two potential sources of biases that the included literatures did not sufficiently address (Table 3). In light of a multicenter case–control study [30], environmental exposure was likely responsible for the incidence of IPF, which has been gradually acknowledged in recent years. Though three studies [8, 9, 24] took this into consideration, unfortunately, only one study matched case and control groups. However, in that single study [9], DM was proven to be the most dangerous factor for IPF in the logistic regression model (OR 4.3, 95% CI 1.9–9.8). Genetic factor, which was considered as one of the covariates according to the guidelines by ATS/ERS [4], was not referred to in any study except one [24]. Additionally, case and control groups in this study were regrettably unmatched. Thus, the impact it had on final conclusions was difficult to evaluate. Publication bias existed (*P* = 0.016 < 0.05) judged by the Egger’s test (Fig. 6).

**Table 3:** Potential sources of bias

Study	Environmental Exposure (IG/CG)		Family IPF (IG/CG)	
Enomoto et al. [7] 2003	Not stated	Not stated	Not stated	Not stated
Miyake et al. [8] 2005	31.7%	8.3%	Not stated	Not stated
Gribbin et al. [10] 2009	Not stated	Not stated	Not stated	Not stated
Ma et al. [9] 2010	dust 56.7% smoke 66.0% chemicals 28.9%	dust 52.1% smoke 69.3% chemicals 21.4%	Not stated	Not stated
Garcia-Sancho et al. [24] 2011	Matched by place of residence	Matched by place of residence	20%	8.7%
Kim et al. [25] 2015	Not stated	Not stated	Not stated	Not stated
Dalleywater et al. [27] 2015	Not stated	Not stated	Not stated	Not stated
Zhong et al. [28] 2016	Not stated	Not stated	Not stated	Not stated
Xu et al. [26] 2020	Not stated	Not stated	Not stated	Not stated

*IG* IPF Group, *CG* Control Group

## Discussion

### Main findings and clinical inspiration

In the present meta-analysis, it was revealed that the prevalence of diabetes was increased markedly in IPF cases compared with controls, which suggests that DM and IPF might be positively associated. However, we noticed that a latest study [31] came to the opposite conclusion. Since all of the included studies are retrospective case–control studies, which are easily affected by recall bias and additionally, the interpretation of the outcome is in the limitation of the significant heterogeneity, which could not be satisfactorily explained, we believe that our conclusion still need further evidence.

Interestingly, a recent review [32] clarified the common features between IPF and pulmonary complications in diabetics. These include clinical characteristics (significant reductions in FVC, FEV1 [33] and DL_CO_ [34–38]), HRCT imaging (the frequently presented UIP pattern [39, 40]) and histopathological changes (thickening of the basal lamina of lung capillaries [41, 42], increased amount of collagen in the alveolar walls [43], etc.), all of which indicated that IPF and diabetes are closely related. This conclusion validated the findings of our meta-analysis as well as equipping them with biological plausibility. However, it is still unclear whether a causal relationship exists between DM and IPF.

Therefore, understanding the exact pathological mechanisms is crucial; namely, how persistent hyperglycemia, a known characteristic of diabetes, gradually contributes to the pulmonary lesions. Studies found that a high glucose concentration could result in nonenzymatic glycation with the ultimate formation of advanced glycation end products (AGEs), which may target type IV collagen in the alveolar basement membrane, thicken the basal lamina both in epithelial and capillary of alveoli and eventually lead to a decrease in pulmonary elasticity and compliance [44–46]. This hypothesis has become recognized as an explanation for the pathological abnormalities of interest, including injured pulmonary function in diabetic individuals. Furthermore, some investigators hold the view that oxidative stress (OS), which refers to an imbalance between free radicals and antioxidants in the body, is intimately connected with the onset of IPF. On one hand, OS can directly enhance nonenzymatic glycation [47], but on another, OS participates in the activation of nuclear factor-kappaB (NF-κB) [48], which is presumably the central part in initiating processes of alveolitis. One study [49] shows that inhibiting the activation of the transcription factor NF-κB could reduce lung injury and fibrosis. Hürdag et al. [50] discovered that OS could decrease superoxide dismutase (SOD), increase nitric oxide synthase (NOS), and contribute to overproduction of nitric oxide (NO) and peroxynitrite (ONOO^−^), potentially giving rise to damage of lung tissue and ultimately pulmonary fibrosis [51, 52]. In addition, inflammatory cytokines play a crucial role, among which transforming growth factor-beta1 (TGF-β1) attracts the most attention. TGF-β1 was found overexpressed in hyperglycemia, which has been documented to promote proliferation and differentiation of fibroblasts, activation of myofibroblasts and deposition of extracellular matrix (ECM) [53–57], all of which will eventually bring about lung fibrosis. In recent years, the relationship between telomere length, DM and IPF has attracted attention. Elevated glucose and increased oxidative stress might interfere with telomerase function, thereby leading to shortened telomere length [58] and a mendelian randomisation study [59] inferred a causal link between shorter telomere length and higher risk of IPF.

Although the possible pathophysiologic mechanisms do explain the disease process, we acknowledge that multiple factors may induce pulmonary fibrosis and that it is also indeterminate to what extend IPF is affected by diabetes. Thus, to solidify the association between DM and IPF, the beneficial effect of antidiabetic therapy should be established. Rangarajan et al. [12] demonstrated that metformin could reverse lung fibrosis in a bleomycin model via AMPK activation, which is also the potential mechanism of metformin in diabetes [60]. The discovery indicated a certain connection between these two diseases and provided a potential evidence on possible benefit of anti-diabetics for the treatment of IPF. Nevertheless, successful therapies in animal models was not particularly efficacious in human studies and a post hoc analysis [16] showed no advantages of metformin when in combination therapy with pirfenidone. Possible explanations may include that AMPK activation is only relevant to certain IPF phenotypes, or insufficient drug concentrations in the lung [61]. Consequently, the effect of antidiabetic treatment in IPF remains uncomfirmed up to now. Since persistent hyperglycemia might participate in the occurrence of pulmonary fibrosis, perhaps future researches could investigate the suitable threshold of blood glucose for IPF and figure out whether timely and effective hypoglycemic therapy would prevent the incidence of the disease. We expect such findings could further strengthen the evidence linking IPF with DM.

### Strengths and limitations

In our meta-analysis, we screened literatures in strict accordance with inclusion and exclusion criteria, designed the study with high quality, and finally demonstrated that DM and IPF are very likely interrelated. Nevertheless, there remain several limitations in our study. Firstly, the heterogeneity was significant, and a reasonable interpretation is still absent. The wide range of prevalence of DM (from 10 to 61% in IPF groups and from 3 to 43% in control groups) in the studies has attracted our attention, which could be responsible for major heterogeneity; we assumed this to be secondary to multiple factors including diagnostic criteria of diabetes (subjective or objective methods) and IPF (differences of diagnostic criteria in different guidelines), selection of populations (with or without underlying diseases), and regional differences. However, the internal causal relationship has not been established to date. Secondly, given that the result is based on case–control studies, which are susceptible to confounding factors, the potential sources of biases have always been a focus. Even though influence of smoking, age, and gender was ruled out in our bias analysis, other covariates such as genetic factor might also cloud the association between DM and IPF, especially considering that at least 30% of patients have predisposing genetic factors which could increase the risk of pulmonary fibrosis [62–64]. Besides, two studies [10, 27] from the UK are based on the THIN database (in Gribbin’s research, IPF cases are from the period 1991–2003 while in Dalleywat’s, cases are from 2000 to 2011), which may be one of the contributory reasons of the notable publication bias, and repeated cases might cause type I errors (false positive conclusions).

## Conclusion

The association between diabetes and IPF was briefly referred to in ATS/ERS clinical practice guideline updated in 2011 [65], but not in the newest edition [4] perhaps owing to the contradiction of existing evidence. Although our study suggested that IPF and DM might be relevant, the causal relationship cannot be completely established up to now. Thus, more evidences are still required.

## Data Availability

All data generated or analysed during this study are included in these published article [7–10, 24–28].

## Appendix

We used Multi-Field Search in medline.

1. (pulmonary fibrosis and diabetes).ti.
2. pulmonary fibrosis.ti. and diabetes.ab.
3. pulmonary fibrosis.ti. and diabetes.tw.

We used Advanced search in Cochrane Library.

1. pulmonary fibrosis in Title Abstract Keyword AND diabetes in Title Abstract Keyword
2. pulmonary fibrosis in Title Abstract Keyword AND diabetes in All Text

We used Quick Search in Embase.

1. 'pulmonary fibrosis':ti AND diabetes:ti,ab,kw
2. 'pulmonary fibrosis':ti AND 'risk factors':ti,ab,kw

We used Advanced search in Web of Science.

1. (TI = (pulmonary fibrosis)) AND TI = (diabetes)
2. (TI = (pulmonary fibrosis)) AND AB = (diabetes)
3. (TI = (pulmonary fibrosis)) AND TS = (diabetes)

We used Advanced search in China National Knowledge Infrastructure.

1. (((idiopathic pulmonary fibrosis[Title]) OR (pulmonary fibrosis[Title])) OR (IPF[Title])) AND (diabetes[Title/Keyword/Abstract])
2. (((idiopathic pulmonary fibrosis[Title]) OR (pulmonary fibrosis[Title])) OR (IPF[Title])) AND (diabetes[Text Word])

## Abbreviations

ATS: American Thoracic Society
DLCO: Diffusion capacity for carbon monoxide of the lung
DM: Diabetes mellitus
ERS: European Respiratory Society
FEV1: Forced expiratory volume in the first second
FVC: Forced vital capcacit
HRCT: High-resolution computed tomography
ILD: Interstitial lung disease
IPF: Idiopathic pulmonary fibrosis
NF-Κb: Nuclear factor-kappaB
NOS: Newcastle–Ottawa Scale
TGF-β1: Transforming growth factor-β1
THIN: The Health Improvement Network
TSA: Trial sequential analysis
UIP: Usual interstitia pneumonia
